# Crop Diversification to Control Rust in Faba Bean Caused by *Uromyces viciae-fabae*

**DOI:** 10.3390/jof9030344

**Published:** 2023-03-11

**Authors:** Ángel M. Villegas-Fernández, Ahmed A. Amarna, Juan Moral, Diego Rubiales

**Affiliations:** 1 Institute for Sustainable Agriculture, CSIC, 14004 Córdoba, Spain; 2 Department of Agronomy, University of Córdoba, 14071 Córdoba, Spain

**Keywords:** intercropping, cultivar mixtures, *Vicia-faba*, rust

## Abstract

*Uromyces viciae-fabae* is a highly specific biotrophic fungus that causes faba bean rust, one of the major diseases affecting this crop. We have assessed the feasibility of using intercropping (faba bean mixed with either pea, wheat or barley) or mixtures of susceptible and resistant cultivars to control rust both under field and controlled conditions. The results of four field intercropping experiments showed a significant reduction in rust severity on faba bean when intercropped with barley (average 22% reduction) but not with the other combinations. This reduction was also confirmed in studies under controlled conditions. The barrier effect of barley appears as the main mechanism explaining rust suppression. Additional experiments under controlled conditions showed that intercropping with barley did not influence the N content of faba bean and that different levels of N nutrition had no impact on rust severity in any case. The cultivar mixture field experiments showed that rust severity in the susceptible cultivar decreased as the proportion of the resistant cultivar in the mixture increased. The importance of the barrier effect of the resistant cultivars was determined in an experiment under controlled conditions. It can be concluded that crop diversification offers great potential to reduce rust in faba bean.

## 1. Introduction

Faba bean (*Vicia-faba* L.) is one of the major legumes grown worldwide [1] both for animal feed and human consumption. Like most cultivated legumes, it is a crop with great potential as a component of sustainable agriculture given its ability to fix atmospheric nitrogen and so become a “natural fertilizer” for the soil or other crops that alternate with it, either in time, space or both [2]. However, the productivity of faba bean may be limited due to its susceptibility to a wide range of phytopathogenic fungi, including the necrotrophic *Botrytis fabae* Sard. (causal agent of Chocolate spot), *Ascochyta fabae* Speg. (causal agent of ascochyta blight) and the biotrophic *Uromyces viciae-fabae* (Pers.) J. Schrot (causal agent of rust) [3]. *U. viciae-fabae* is an autoecious macrocyclic species not requiring an alternate host to complete its lifecycle, and sexual reproduction is not commonly observed in temperate regions. Urediospores are dispersed mainly by wind and infect the aerial parts of the plant (leaves, stems and pods). Yield losses due to rust may reach a 20% yield reduction [4], although it has been reported in some cases to reach even 70% [5]. The use of fungicides may achieve effective rust control, although it is expensive for the farmer and not environmentally desirable [6]. As for genetic resistance, some sources of resistance have been described [7,8,9], but few commercial varieties with complete resistance have been released so far [5]. 

Crop diversification has proven to be helpful in controlling fungal pathogens in different crops. This has been shown by mixing different crops in the same field or by growing together cultivars of the same crop with differential responses to the pathogen. Reductions in disease severity of 20–40% have been described for both strategies in several crops [10,11], although the level of control may differ from one situation to another, depending on a wide range of factors such as crops to combine, intercrop pattern, crop cultivars or weather conditions. Different mechanisms have been suggested to explain this impact of diversification on fungal pathogens, ranging from barrier effects or microclimatic modifications to enhancement of the crops due to more efficient use of resources [10,12]. In addition, nitrogen nutrition may also play a role in disease suppression by diversification in some cases, although its mechanisms are not clear yet [13,14].

With regard to faba bean, reports of disease suppression by intercropping with cereals include chocolate spot [10,13,15], fusarium wilt [16,17], rhizoctonia [18] and rust [19,20,21]. The use of cultivar mixtures for reducing disease incidence in faba bean has been less studied, with no reports available so far.

The main objective of this work was to assess the reduction in rust in faba bean either by alternate intercropping with different crops (pea, wheat and barley) or using a mixture of two cultivars of faba bean, one susceptible and one resistant to the disease. Additionally, the mechanisms behind the different interactions in these mixtures, including the effect of nitrogen nutrition, were investigated through several experiments under controlled conditions.

## 2. Materials and Methods

### 2.1. Field Trials

**Figure 1:**
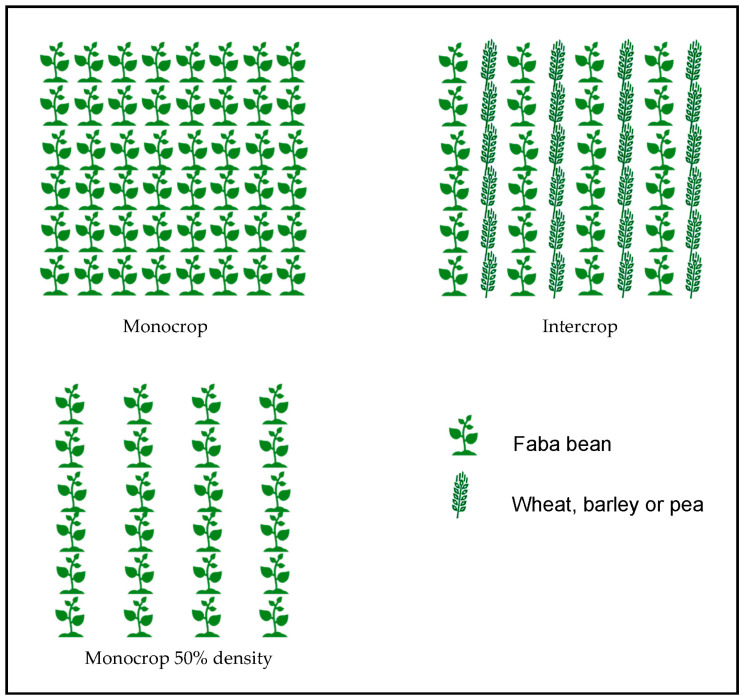
Experimental design for the intercropping experiments.

From 2015 to 2019, four field trials aiming to study the effect of intercropping on faba bean rust were conducted in two sites (Córdoba and Almodóvar del Rio) located in the South of Spain (Table 1). Faba bean cv. Baraca was intercropped with either pea (cv. Messire), durum wheat (cv. Califa) or barley (cv. Henley). Monocrops of these four cultivars were also included, plus a monocrop of faba bean at 50% of sowing density (doubling the distance between rows). Plot size was 2.8 m × 3 m (8 rows per plot at 35 cm distance between them). Legumes were sown at a density of 80 seeds/m^2^ and cereals at 200 seeds/m^2^. Alternate intercropping with replacement at 50% was used, i.e., a row of each crop is alternatively sown to a final rate of 50/50 (Figure 1). The experiment design was a randomized complete block with four replications.

Rust disease severity (DS) was visually estimated as the percentage of the whole plant canopy showing rust pustules. The severity value for each plot was that of the overall DS on the two middle rows (discarding those plants at the extremes of the rows), so to avoid any edge effect. Evaluations started one week after the appearance of the first symptoms and were repeated every 7–10 days until plant senescence. 

Yield and biomass were obtained by harvesting two rows of each crop per plot. However, not all crops could be harvested in all trials due to different problems (e.g., bird damage on the cereals). Grain yield for faba bean was determined in trials IC-Crd-16, IC-Crd-18 and IC-Crd-19; for wheat and barley in trial IC-Crd-19 (where cereal plants were covered with a mesh at maturity to avoid bird damage) and for pea in trials IC-Crd-16 and IC-Crd-18. Crop biomass was determined for trial IC-Crd-19 by drying plants in an oven at 60 °C for three days, weighing them and then, after threshing, subtracting grain weight. Crop height was assessed at full maturity in trials IC-Crd-18 and IC-Crd-19, measuring five plants per plot with a ruler without stretching them. 

The Land Equivalent Ratios (LER) of grain or biomass yields were calculated, when possible, as follows [22]:

$$\mathrm{LERpx}=\frac{Yip}{\mathrm{Ymp}}+\frac{Yix}{\mathrm{Ymx}}$$

where LERpx represents the LER value of a given combination of faba bean and another crop “x” (i.e., pea, wheat or barley). The Y_ip_ and Y_mp_ parameters are the yields (grain or biomass) of faba bean intercropped and monocrop, respectively; Y_ix_ and Y_mx_ are the yields (grain or biomass) of the other crops in intercrop or monocrop, respectively.

**Figure 2:**
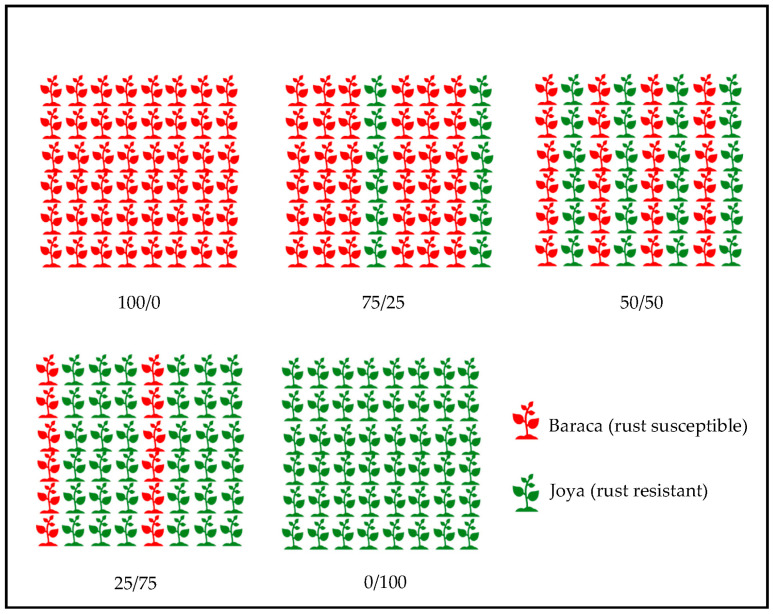
Experimental design for cultivar mixture experiments of two cultivars of faba bean with different degrees of susceptibility to rust (*Uromyces vicia-fabae*): cv. Baraca (susceptible) and cv. Joya (resistant).

Additionally, four trials for cultivar mixtures were performed in 2015–2019 (Table 2). Faba bean cv. Baraca (susceptible to rust) was mixed with resistant cultivar Joya at different proportions (Baraca/Joya: 100/0, 75/25, 50/50, 25/75 and 0/100), in alternate replacement intercropping (Figure 2). Faba bean cv. Joya is similar in morphology and growth cycle to Baraca, differing in the rust resistance, as it is derived from a cross between Baraca with the resistant accessions VF1273. The disease severity (DS) of rust in Baraca was evaluated in the same way as previously described. Grain yields were determined for trials VM-Crd-15 and VM-Crd-19, and plant biomass for trial VM-Crd-19. Plant height was recorded as described before in trials VM-Crd-18 and VM-Crd-19.

### 2.2. Controlled Condition Experiments

Four experiments on seedlings under controlled conditions were carried out to clarify the mechanisms responsible for the fungal disease reductions in the interaction between the different crops. All experiments were run in completely randomized blocks. The *Uromyces viciae-fabae* isolate CO-07 was used for plant inoculations. This isolate is part of the faba bean rust isolate collection at IAS-CSIC (Córdoba). Originally, it was isolated from naturally-infected faba bean plants growing in a field in Córdoba. Urediospores were taken from a single pustule and inoculated on a healthy plant under controlled conditions in a growing chamber to produce a monopustular isolate. Growing conditions were: photoperiod of 12 h of visible light (150 μmol m^−2^ s^−1^ photon flux density) at 25 °C and 12 h of darkness at 20 °C. Urediospores were subsequently collected in plastic tubes after aspiring them with the help of a vacuum pump. Then, they were inoculated on faba bean plants to multiply the isolate under the same conditions. Urediospores were again collected and kept in plastic tubes in a freezer at −80 °C. 

**Figure 3:**
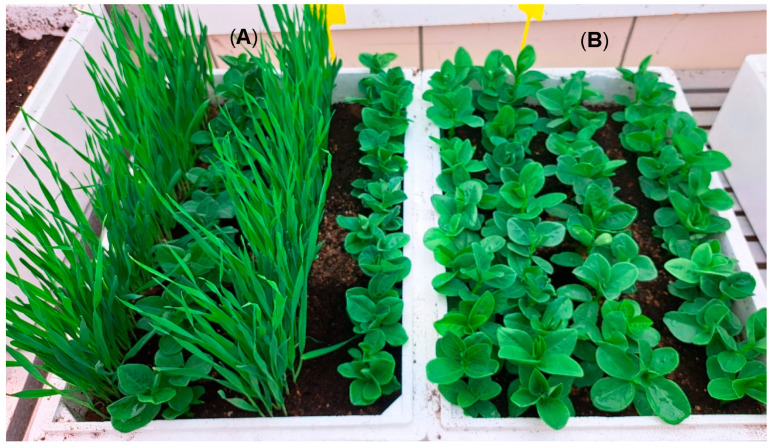
Faba bean cv. Baraca intercropped with barley cv. Henley (**A**) and monocropped (**B**) in the first experiment under controlled conditions for *Uromyces viciae-fabae* management.

In the first experiment, the differential behavior of barley and wheat as barriers to rust spore dispersal was assessed. To this end, polystyrene boxes (34 × 55 × 16 cm, width:length:height) were filled with a mixture of sand and peat at a 1:3 rate (*v*:*v*) and placed in a growth chamber. Four rows of seeds were sown in each box for three treatments: faba bean monocrop, faba bean/wheat and faba bean/barley intercrops, with five replications (one treatment in each box, fifteen boxes in total). The distance between rows was 7 cm, and the number of seeds per row was 18 for faba bean and 150 for the cereals. The first row was always a cereal in the intercrops, with faba bean in the second and fourth rows (Figure 3). Growing conditions were the same as described above for the multiplication of the isolate. Seventeen days after sowing, faba bean seedlings were inoculated by dusting them with a mixture of rust urediospores and pure talc (1:10), totaling 3 mg of spores per plant. The pathogen spores were homogenously sprayed on the first row of plants perpendicularly to the side of the box. The number of cereal plant leaves was counted at the time of inoculation. Inoculated plants were incubated for 24 h in darkness at 20 °C and 90% relative humidity. Then, growing conditions were reinstated. Fifteen days after inoculation, disease severity was evaluated in faba bean plants in the second and fourth row of each box as follows: three infected leaves per plant were randomly chosen, and the number of pustules in an equally randomly selected area of 1 cm^2^ were counted in each of them, then averaged; the number of infected leaves and the total number of leaves per plant were also counted, and their proportion calculated; finally, the average number of pustules per leaf was calculated multiplying the average number of pustules per infected leaf by the proportion of infected leaves in the plant. Cereal plants were subsequently pulled out, dried in an oven at 60 °C for 3 days, and then weighed to determine biomass.

In the second and third experiments, the possible interaction of nitrogen nutrition with the intercropping system and rust disease severity was investigated. The experiments were carried out similarly to the previous one, inside a chamber in identical growth conditions. In these experiments, the dimensions of the polystyrene boxes were 27.5 × 34 × 16 cm (width:length:height), so the number of seeds varied: 11 for faba bean and 90 for cereals. For the second experiment, six treatments were included: faba bean monocrop and intercrop of faba bean with barley, each with three levels of nitrogen nutrition. The nitrogen nutrition was performed using irrigation solutions at three different levels: N0, N1 and N2, which were 0, 750 and 1500 mg/L of ammonium nitrate, respectively. Plants were irrigated on demand with one liter of their respective solution per box, twice before inoculation. Treatments were replicated five times. The plant inoculation was conducted as in the first experiment but performed from above the box, perpendicular to the soil, to avoid any barrier effect. Rust severity on faba bean plants was evaluated twelve days after inoculation, calculating the average number of pustules per leaf as in the previous experiment. The objective of the third experiment was to investigate any possible effect of intercropping on the nutrient content of faba bean leaves. It consisted of two treatments, faba bean monocrop and faba bean intercropped with barley, with four replications. No fertilization was added. Twelve days after sowing, the leaves of faba bean plants were detached and frozen until they were taken for analysis of foliar nitrogen content. Nitrogen content was determined by the Kjeldahl method [23].

**Figure 4:**
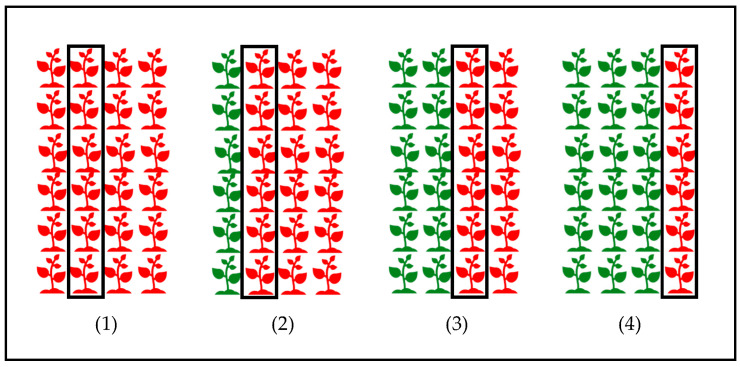
Experimental design for the cultivar mixture experiment in growth chambers under controlled conditions. The susceptible cultivar is Baraca (red), and the resistant one is Joya (green). The four treatments appear numbered: (1) all rows of cv. Baraca, the first one acting as a barrier; (2) one row of cv. Joya acting as a barrier, and three rows of cv. Baraca; (3) two rows of cv. Joya acting as barrier, and two rows of cv. Baraca; (4) three rows of cv. Joya as barrier, and one row of cv. Baraca. The squared rows are those that were evaluated for rust.

Finally, the fourth experiment aimed to assess the barrier effect of resistant faba bean cv. Joya on the reduction in rust on susceptible cv. Baraca in the cultivar mixtures field trials. Again, seeds were sown on the smaller polystyrene boxes described above, and four treatments were studied (Figure 4). Growth conditions and inoculation were the same as in the first experiment. Inoculation was performed perpendicularly to the first row, which was sown with the cv. Joya in the three treatments with cultivar mixtures. Disease evaluation was performed as in the previous experiments 12 days after inoculation only for the rows shown in Figure 4. Plant height was determined at the time of inoculation. 

### 2.3. Statistical Analysis

The effect of the various treatments (i.e., different intercropping) on the final disease severity (% leaves covered by pustules) was studied using a Mixed-Effects Model using the treatment as a fixed factor and the environment (defined as the combination of plot-year) as a random factor. The treatment means were compared using Tukey’s test at *p =* 0.05. Likewise, a General Linear Model was performed to study the effects of the treatments (intercropping or nitrogen levels) on the rust severity under controlled conditions, followed by Tukey’s test. To test whether the LER value of each treatment differed from the hypothesized value (µ ≠ 1), the confidential interval (C.I.) for the mean of each treatment was calculated, and a one-sample *t*-test was performed. Two-way ANOVA was used to study the effects of the crop and cultivation system (intercrop or monocrop) on biomass production. Subsequently, biomass means were compared by Fisher’s protected Least Significant Differences Test (LSD at *p* < 0.05). The effect of the cultivar showing ratio in the crop mixtures was studied by employing the exponential equation of [24]:

∂y/(∂r) = −by

where y represents the severity of symptoms on the susceptible cultivar (SAUDPC), r represents the ratio of the resistant cultivar and b is the rate of decrease in disease per unit increase in the resistant cultivar (i.e., slope). Overall, there was a higher effect with higher disease severity due to the resistant cultivar. The previous equation was linearized as Lny = a − b × r, in which a is a constant. The linearized equation fits the data of the different seasons. It was selected based on the coefficient of determination (R^2^ > 0.450; *p* < 0.001) and the pattern of residuals and was compared with other exponential models [25]. 

In the experiments conducted under controlled conditions, disease severity was quantified as the number of rust pustules per leaf. A Generalized Linear Model (GLM) was performed to study the treatment significance. When necessary, this dependent variable was arcsine-transformed for variance homogeneity. After GLM, we compared the means by Fisher’s protected LSD at *p <* 0.05. Statistical analyses were performed using SPSS version 23.0 for Windows.

## 3. Results

### 3.1. Intercropping in the System Faba Bean/Rust 

#### 3.1.1. Field Trials

**Figure 5:**
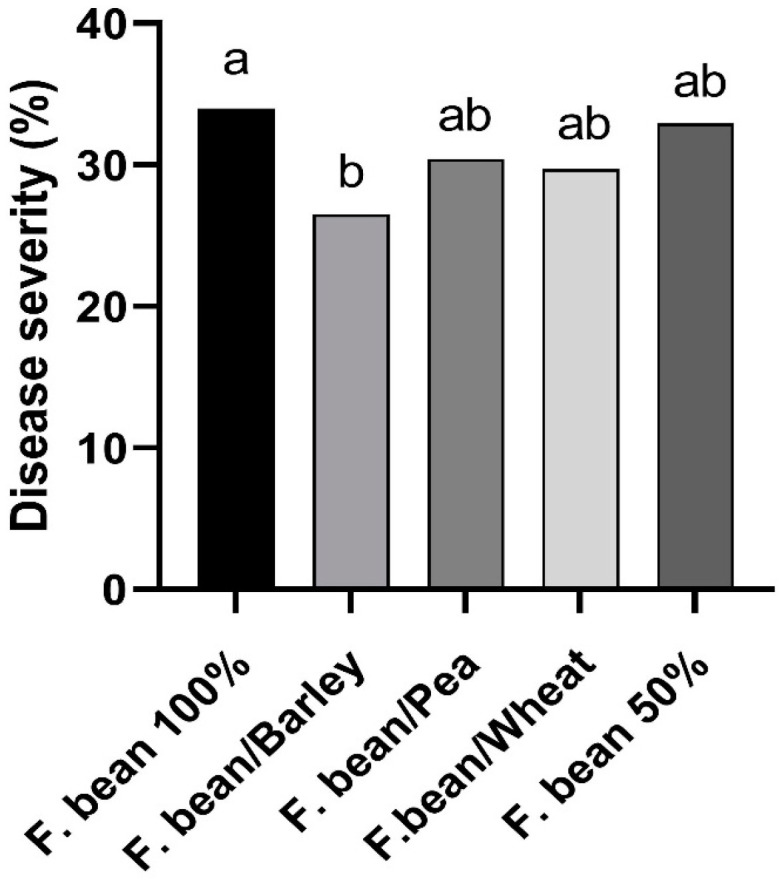
Disease severity of rust (caused by *Uromyces viciae-fabae*) in faba bean in the different treatments evaluated across the four intercropping field trials. Different letters mean significant differences (Friedman’s test, *p* < 0.05).

Disease severity (DS) varied with the environment, being highest on faba bean monocropped at IC-Crd-16 (82%) and the lowest at IC-Crd-19 (6.7%) (Table 3). In most cases, crop combinations brought about a reduction in DS as compared to the monocrop, although with differences between the intercropping types. The analysis across all four trials (Figure 5) showed that the combination of faba bean with barley resulted in the lowest level of rust disease severity (26.5%) as compared to the severity in the faba bean monocrop (34%), which means a significant (*p* < 0.05) average reduction of 22%. 

In order to study the effect of intercropping on the grain and biomass yield, we calculated the LER ratios (intercrop yield: monocrop yields). Thus, in the theoretical case of intercropping having no impact on the yields of the crops, LER should equal approximately 1. In our study, LER values for grain and biomass yields did not significantly deviate from 1, i.e., intercropping did not negatively or positively impact faba bean yield or biomass (Table 4). 

**Figure 6:**
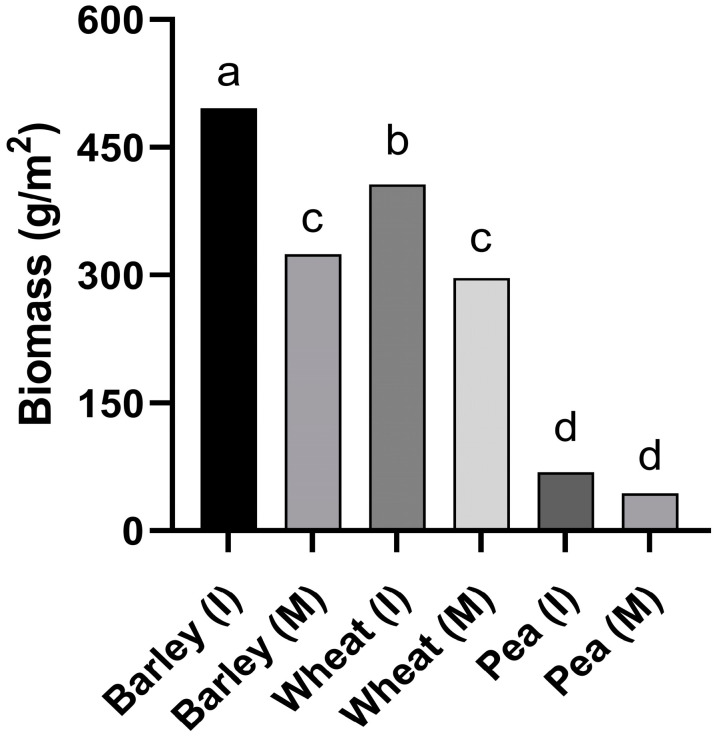
Biomass (g/m^2^) for the different companion crops (I: intercrop; M: monocrop) in trial IC-Crd-19. Different letters mean significant differences (LSD test, *p* < 0.05).

However, a significant interaction between crop and cultivation system (intercrop or monocrop) was found in the factorial analysis for biomass per row of the companion crops (*p* < 0.05): barley intercropped with faba bean presented the highest biomass per row of all treatments, including barley monocrop; the biomass of wheat intercropped with faba bean was higher than that of wheat monocrop, while pea biomass (intercrop and monocrop) was the lowest of all treatments (Figure 6).

The plant height differences between faba bean and their companion crops were determined, resulting in the combination of faba bean with barley presenting the lowest difference, 0.17 cm; in comparison, the differences between faba bean and wheat or pea were similar (*p* < 0.05) and higher than 13 cm.

#### 3.1.2. Controlled Conditions Experiments

In the first experiment, treatments and position row (distance to the point of inoculation) significantly (*p* < 0.05) affected the number of rust pustules per leaf but no interaction between them was detected. The highest infection was quantified on faba bean growing in monocrop (average = 3.15 pustules/leaf). This was reduced on faba bean intercropped with wheat (2.14 pustules/leaf) and further reduced on faba bean intercropped with barley (1.15 pustules/leaf). Leaves of those plants in the row further from the inoculation point presented about half of the pustules as those in the second row (7.8 vs. 3.7 pustules/leaf). The dried biomass and the average number of leaves per plant of barley were higher than those of wheat (*p* < 0.05): 15.5 g and 5.3 leaves/plant vs. 9.02 g and 4.5 leaves/plant.

In the second experiment under controlled conditions, the statistical analysis revealed no significant differences in the number of pustules per leaf for any of the three factors under study, that is, nitrogen level (N0, N1 or N2), cropping system (monocrop or intercrop) or row in the tray (Table 5). Similarly, no significant difference was found in the third experiment for nitrogen content in faba bean leaves either in monocrop (5.78% N content) or intercropped with barley (6.15% N content).

### 3.2. Cultivar Mixtures in the System Faba Bean/Rust

#### 3.2.1. Field Trials

**Figure 7:**
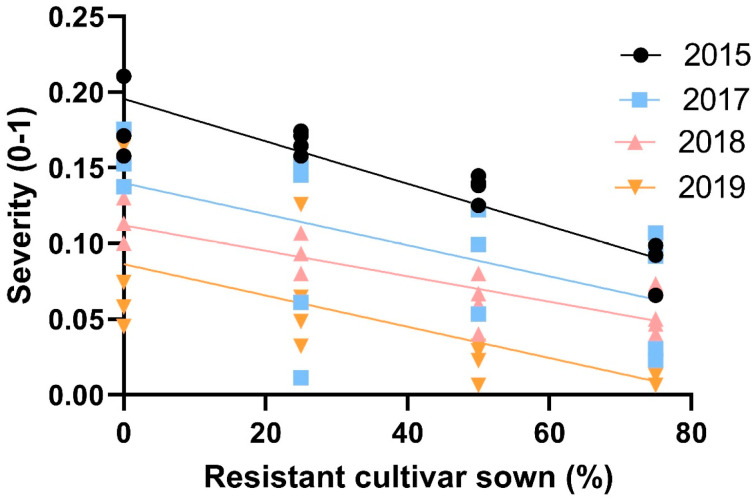
Linear regression between the rust disease severity (linearized as natural logarithm), caused by *Uromyces viciae-fabae* in susceptible faba bean cv. Baraca and the proportions of resistant cv. Joya in four field experiments (in all the cases: R^2^ > 0.450; *p* < 0.001).

Rust levels in the different trials covered a span of low to medium infection pressure (Table 6). Disease severity values linearly decreased in susceptible cv. Baraca as the proportion of resistant cv. Joya increased in each experiment, fitting significantly (*p* < 0.001) a linear regression (Figure 7).

No significant differences were detected in yield for any of the different cultivar combinations. However, results did show that cv. Joya has a significantly higher biomass and is 24 cm taller than cv. Baraca (Table 7).

#### 3.2.2. Controlled Conditions Experiment

The analysis of the experiment under controlled conditions showed significant differences in the number of pustules per leaf in plants of cv. Baraca depending on whether the previous row consisted of Joya or Baraca: those plants with Joya before them presented less disease incidence (9 vs 12.8 pustules per leaf, *p* < 0.05). On the contrary, the number of rows before the evaluated plants did not appear to influence disease severity. As for plant height, Joya turned out to be nearly 1 cm taller than Baraca (13.1 vs. 12.2 cm, *p* < 0.05).

## 4. Discussion

In this work, we have assessed the impact of intercropping and cultivar mixtures on the system faba bean-*Uromyces viciae-fabae*, attaining significant reductions in the disease with both systems. Field trials for intercropping found an overall reduction in rust severity of 22% for the combination of faba bean with barley. No effect could be established for any of the other combinations. Recently Shtaya et al. [19] also identified the mixture of faba bean and barley as the most effective in reducing rust (35% in their case) over mixtures with other cereals. Other authors, Guo et al. [20] and Luo et al. [21], only tested faba bean mixed with wheat and obtained rust reductions in the range of 20–50% for the different treatments they tested. On the contrary, Kamalongo and Cannon [26] did not find rust reduction in faba bean combined with wheat. Nevertheless, it is not easy to compare these studies, as the experimental designs differed greatly. We studied rust suppression on faba bean using alternate replacement intercropping, finding that barley appears to be a more effective accompanying crop to suppress rust infection than wheat. Shtaya et al. [19] used mixed intercropping, and Guo et al. [20] and Luo et al. [21] used strip intercropping with a high number of wheat rows separating the rows of faba bean. Kamalongo and Cannon [26] did study alternate replacement for the combination of faba bean and wheat, and their results confirm ours. It can be concluded that faba bean with barley appears to be more reliable in controlling rust than other mixtures.

The combination of faba bean with barley has also been described as effective against *Botrytis fabae*, a causal agent of chocolate spot [15]. Barley also appears as a good partner for other legumes for disease suppression, as it has been reported to help to control *Erysiphe pisi*, the causal agent of powdery mildew, in pea [27]; fungal species (*Ascochyta pisi*, *Mycosphaerella pinodes*, and *Phoma pinodella*) causing ascochyta blight in pea [28]; and *Pleiochaeta setosa* causing brown spot in lupine [29].

Several mechanisms have been suggested to explain the reduction in diseases with intercropping [10]: inoculum dilution effect by the decreased density of the host crop; barrier effect by the added crop to spore dispersion; morphological and physiological changes in the host; changes in the microclimate that make it less favourable for fungal dispersion progression; inhibition of the fungal infection by allelochemicals. In the system faba bean-*U. viciae-fabae*, Shtaya et al. [19] have recently suggested that the reduction might be due to a barrier effect. However, given their experimental design (mixed intercropping), other mechanisms such as inoculum dilution or altered microenvironment may play a greater role. Likewise, Guo et al. [20] and Luo et al. [21] have found variations in the plant microenvironment when intercropped (towards higher humidity and temperature) that might account for at least some part of the decrease, but, considering the experimental design (strip intercropping), it is quite likely that there is a significant barrier effect.

Our results point to a barrier effect, which would be expected given the alternate intercropping system that has been employed. The results of the faba bean at half density support this: faba bean plants are at double the distance than in the normal monocrop, which in principle would render a dilution of inoculum and less humidity due to higher aeration, all of which should lead to a hampering of disease progress and less incidence and severity. However, the disease levels are equal to those of the treatment of monocrop at a normal distance. It is the introduction of a different crop in between the rows of faba bean that hampers infection. 

The higher biomass and height of barley as compared to the two other companion crops (wheat and pea) in the field trials might explain why barley performs better as a barrier. In the experiment under controlled conditions, we tested the performances of barley and wheat as barriers, confirming that barley is the most efficient barrier. In this case, the biomass and height of the plantlets of barley were also higher than those of wheat plants. The fact that barley has a more vigorous development than wheat has been previously described [30,31,32], and our results show that this vigour makes barley a better candidate than wheat as an intercropping partner to control rust in faba bean.

It has been reported that the role of nitrogen in plants’ response to fungal infection is diverse, with positive, negative or neutral effects on disease development [33,34,35]. We studied the impact of nitrogen with two experiments under controlled conditions. In the first of them, no difference was detected in damages on faba bean caused by *U. viciae-fabae* for any of the fertilisation levels tested. This is in contrast with the works of Guo et al. [20] and Luo et al. [21], both reporting that disease increased with N fertilization, which was related to changes in the microenvironment in the first study; the conditions of our experiment, with young plants evaluated over a short period, might account for this difference. It is remarkable, however, that there were no differences in disease levels between monocropped and intercropped faba beans in this experiment, where inoculation had been performed from the top. This appears to confirm the importance of the barrier effect for the control of rust in intercropping. In the second experiment, it was found that intercropping with barley does not alter the nitrogen content in faba bean leaves, which would also disregard a potential effect of intercropping on nitrogen content as a mechanism to reduce rust. 

The use of cultivar mixtures to control crop diseases has been very limited and restricted mostly to cereals, mainly wheat [36,37,38,39,40]. For legumes, only a few studies have assessed their effectiveness in controlling anthracnose and rust in common beans [41,42] or powdery mildew in pea [27]. These mixtures appear to avoid the surmounting of pathogens’ resistance by reducing the high selection pressure that homogeneous monocultivar crops impose on them [43]. The key issue is to mix cultivars with resistance and susceptibility to the disease in such a proportion that disease severity is kept to acceptable levels, while the resistance is prevented from being broken over. The mechanisms that explain disease reduction are quite similar to those already mentioned for intercropping, although the dilution of inoculum appears, in principle, as the most relevant [11]. Our results show that in the case of the system faba bean/rust, the use of cultivar mixtures may be effective. The fact that the resistant cultivar Joya grew higher and produced more biomass than the susceptible cultivar Baraca raised the question of the importance of the barrier effect in addition to the dilution of inoculum. The differential response of both cultivars when standing in the way of inoculation in the experiment under controlled conditions confirmed that the barrier effect plays an essential role in reducing rust in this particular mixture. This stresses the importance of choosing partners for mixtures based on their contrasting responses to the disease and other factors, such as plant architecture [44], that may provide additional advantages.

In conclusion, in this work, it has been established that the combination of barley and faba bean in alternate intercropping is an excellent tool to help control rust in faba bean and that the barrier effect by the cereal is the main mechanism operating in this situation. Equally, it is the first time that it has been proved that cultivar mixtures in faba bean may be effective in controlling rust. Further work should focus on determining the features that mixed cultivars should possess for optimum performance in a particular environment either for intercropping or cultivar mixtures.

## Figures and Tables

**Table 1:** Field trials conducted for the study of the effect of intercropping on the system faba bean-*Uromyces viciae-fabae*.

Trial	IC-Crd-16	IC-Crd-18	IC-Alm-18	IC-Crd-19
Location	Córdoba	Córdoba	Almodóvar	Córdoba
Season	2015/16	2017/18	2017/18	2018/19
Max. T (°C)	31.7	29.7	30.6	35.7
Min. T (°C)	−2.3	−3.4	−3.2	−2.8
Mean T (°C)	12.6	12.0	12.7	12.5
Rain (ml)	336	444.6	422	110.4

**Table 2:** Field trials carried out for the study of the effect of cultivar mixtures on the system faba bean-*Uromyces viciae-fabae*.

Trial	VM-Crd-15	VM-Crd-17	VM-Crd-18	VM-Crd-19
Location	Córdoba	Córdoba	Córdoba	Córdoba
Season	2014/15	2016/17	2017/18	2018/19
Max. T (°C)	33.1	32.2	29.7	35.7
Min. T (°C)	−3.3	−3.4	−3.4	−2.8
Mean T (°C)	11.5	12.1	12.0	12.5
Rain (ml)	155.6	213.6	444.6	110.4

**Table 3:** Final disease severity (DS, evaluated as the percentage of the whole faba bean plant canopy covered by rust (caused by *Uromyces viciae-fabae*) for each treatment of the different intercropping trials carried out (SE: standard error).

	IC-Crd-16	IC-Crd-18	IC-Alm-18	IC-Crd-19
Faba bean 100%	82.0	13.5	33.7	6.7
Faba bean/barley	68.3	12.1	24.0	1.6
Faba bean/pea	80.6	13.0	24.7	3.4
Faba bean/wheat	74.3	15.9	25.9	2.7
Faba bean 50%	66.5	19.5	39.0	6.6
SE	2.8	2.2	2.8	1.2

**Table 4:** LER values for grain yield (trial IC-Crd-19 for the combinations of faba bean with barley or wheat, and trials IC-Crd-16 and IC-Crd-18 for the combination of faba bean with pea) and biomass (trial IC-Crd-19). No significant differences between any of them in each case were detected, and they did not significantly deviate from 1.

	LER Grain Yield	LER Biomass
Faba bean/barley	0.89	1.01
Faba bean/wheat	0.95	1.02
Faba bean/pea	0.85	1.44

**Table 5:** Pustules per leaf in faba bean plants grown in monocrop or intercropped with barley at three nitrogen fertilization levels (N0, N1 and N2) and for row position in the tray (2 or 4). No significant differences were found for any of the tested factors.

	N0 (0 mg/L)		N1 (750 mg/L)		N2 (1500 mg/L)	
	row 2	row 4	row 2	row 4	row 2	row 4
Faba bean	3.6	3.3	3.5	4.8	3.6	3.1
Faba bean/barley	3.3	3.5	3.5	4.6	3.4	2.5

**Table 6:** Final rust severity (DS) for each treatment of the different intercropping trials conducted by mixing the cultivars Baraca (Susceptible) and Joya (Resistant).

S/R in Mixture (%) ^a^	VM-Crd-15 ^b^	VM-Crd-17	VM-Crd-18	VM-Crd-19
100/0	22.4	19.7	16.6	13.2
75/25	19.0	12.1	14.5	10.5
50/50	15.3	13.0	9.2	3.5
25/75	9.4	8.2	7.9	2.5
SE ^c^	1.1	2.2	1.0	2.2

^a^ S/R: proportions of susceptible cultivar and resistant cultivar sown; ^b^ Disease severity was evaluated in cultivar Baraca. ^c^ SE: standard error.

**Table 7:** Biomass (VM-Crd-19) and plant height (VM-Crd-18 and VM-Crd-19) for the two cultivars tested in the cultivar mixtures experiments. Different letters mean significant differences (LSD test, *p* < 0.05).

Cultivar	Biomass (g/m^2^)	Height (cm)
Joya	524.1 ^a^	98.2 ^a^
Baraca	321.0 ^b^	74.1 ^b^

## Data Availability

All relevant data are within the paper.
